# Supplementary material for: Rapid turnover of life-cycle-related genes in the brown algae
Source: Genome Biol. 2019 Feb 14;20:35. doi: 10.1186/s13059-019-1630-6 (PMC6374913; doi:10.1186/s13059-019-1630-6)
Supplement: Supplementary file 3 — Protein alignments, corresponding Gblocks html file and CDS sequences for the lineage conserved gametophyte-biased genes. (ZIP 2109 kb) [file 13059_2019_1630_MOESM3_ESM.zip › Con_GA_alignments/Ec-28_000090.aa_ali.fasta-gb.htm]

Ec-28\_000090.aa.fasta.trans.aa\_ali.fasta


## Gblocks 0.91b Results

Processed file: **Ec-28\_000090.aa.fasta.trans.aa\_ali.fasta**  
Number of sequences: **2**  
Alignment assumed to be: **Protein**  
New number of positions: **327** (selected positions are underlined in blue)

```
                         10        20        30        40        50        60
                 =========+=========+=========+=========+=========+=========+
Ec-28_000090     MELEKQGVALKAASAMLKGGKTADKSLFRRGSKRLSNEGVDGALNAKHLDEIKKAEHLKK
MSTRG.20771.1    ------------------------------------------------------------
                                                                             


                         70        80        90       100       110       120
                 =========+=========+=========+=========+=========+=========+
Ec-28_000090     KLKAHHDEARAVLAKKLQVENARTGRVIKTNLGKYANPKLAQGAKAAVLASVVEYLNGEE
MSTRG.20771.1    ------------------------------------------------------------
                                                                             


                        130       140       150       160       170       180
                 =========+=========+=========+=========+=========+=========+
Ec-28_000090     FVRIVAAPLNYQSPDFVRAMEAESGVMEISFSPTNARQRSMASLPVGAPDYDSKMALLQE
MSTRG.20771.1    ------------------------------------------------------------
                                                                             


                        190       200       210       220       230       240
                 =========+=========+=========+=========+=========+=========+
Ec-28_000090     QKAIEQQQRMEEEALRRQEMLERENKAAMEQDAADALAMANAANQRRLEEMQRQQDEELE
MSTRG.20771.1    ------------------------------------------------------------
                                                                             


                        250       260       270       280       290       300
                 =========+=========+=========+=========+=========+=========+
Ec-28_000090     EQRRQLDEHQEHLRREAELARMRHKEELDLHRAEMEREREAVEARQAEAKRLQEEELEKQ
MSTRG.20771.1    ------------------------------------------------------------
                                                                             


                        310       320       330       340       350       360
                 =========+=========+=========+=========+=========+=========+
Ec-28_000090     RQEMRRKHEEDMELQRQELARHAEEVARHKAELAKHNEGVGLENGVLTSQSDSPGVVHHQ
MSTRG.20771.1    ------------------------------------------------------------
                                                                             


                        370       380       390       400       410       420
                 =========+=========+=========+=========+=========+=========+
Ec-28_000090     QEARLEKEALEEMRRRENEAEEDERRRLQEAVAKHQEETRLEAERVKAELEALKLEREAV
MSTRG.20771.1    ------------------------------------------------------------
                                                                             


                        430       440       450       460       470       480
                 =========+=========+=========+=========+=========+=========+
Ec-28_000090     AQAEKAEREAEEEARRAREAEAAAEALRIEQENDRRKASEVAMRAQIEKELEERVARDIQ
MSTRG.20771.1    ------------------------------------------------------------
                                                                             


                        490       500       510       520       530       540
                 =========+=========+=========+=========+=========+=========+
Ec-28_000090     IERDRVAAESAALQAKMKEFEEEREKAALRAAAEVEAQKAAEAALVARERAAAESLALGR
MSTRG.20771.1    ------------------------------------------------------------
                                                                             


                        550       560       570       580       590       600
                 =========+=========+=========+=========+=========+=========+
Ec-28_000090     ALSAKADLDATTASKEGLSSDRRDPSGREAGTAPVPIGYLVGDNVQALCTPVGGATGSKE
MSTRG.20771.1    ------------------------------------------------------------
                                                                             


                        610       620       630       640       650       660
                 =========+=========+=========+=========+=========+=========+
Ec-28_000090     YISGEVAHVNPDGSLDVILADGRMRQEVPIAEIKFVSRSEPPSPAGSSAGEQSFWDGNEQ
MSTRG.20771.1    ------------------------------------------------------------
                                                                             


                        670       680       690       700       710       720
                 =========+=========+=========+=========+=========+=========+
Ec-28_000090     GSESSAGQNSVRRGGGDGAAAAAAAAAAVRRRKRKRRSGILVVIIIINRSRSRSRIIIIN
MSTRG.20771.1    ------------------------------------------------------------
                                                                             


                        730       740       750       760       770       780
                 =========+=========+=========+=========+=========+=========+
Ec-28_000090     DDDDVVVVVVVVAGGGGGGGGGGGGGKASTMFAPGVVRRVHEDGSADVELETGSVLLQRS
MSTRG.20771.1    ------------------------------------------------------------
                                                                             


                        790       800       810       820       830       840
                 =========+=========+=========+=========+=========+=========+
Ec-28_000090     AREVRVVWKGKPSVTSSSLSLPSSLSSSSPSPMSSSSPGSSSSPLALGDLAVGDRVEARY
MSTRG.20771.1    ------------------------------------------------------------
                                                                             


                        850       860       870       880       890       900
                 =========+=========+=========+=========+=========+=========+
Ec-28_000090     AGEPEFYPGVVTSTEDGVSVNFDKGWHGASIPLKDVRRPNVKPGRSRDGKPSTLSAGARL
MSTRG.20771.1    ------------------------------------------------------------
                                                                             


                        910       920       930       940       950       960
                 =========+=========+=========+=========+=========+=========+
Ec-28_000090     SSTGCILDLDVGSLGGDTFAVGDQVQFRLTLGLRAGPPQTHNGVIRTAHPNGSYLVGFED
MSTRG.20771.1    ------------------------------------------------------------
                                                                             


                        970       980       990      1000      1010      1020
                 =========+=========+=========+=========+=========+=========+
Ec-28_000090     GTTRDQVQVRSLTGFDWTPHGSEAYRRAATDTSQATTAVAAAACPGQGRGGGGGSHGATT
MSTRG.20771.1    ------------------------------------------------------------
                                                                             


                       1030      1040      1050      1060      1070      1080
                 =========+=========+=========+=========+=========+=========+
Ec-28_000090     GAGAVSPLDSGKPSKGPPRGSMFDRFGFPRKVFPDGGGLGGTTGGGGGGSVLQRRATHGG
MSTRG.20771.1    ------------------------------------------------------------
                                                                             


                       1090      1100      1110      1120      1130      1140
                 =========+=========+=========+=========+=========+=========+
Ec-28_000090     VVDSGEPGLRVDVNMLHFRSQFMDPDTAAQAILTPNNAGMFRPHVIGRGGGGGRGGHRQT
MSTRG.20771.1    ------------------------------------------------------------
                                                                             


                       1150      1160      1170      1180      1190      1200
                 =========+=========+=========+=========+=========+=========+
Ec-28_000090     ASELSYNSTTDESDADRDEDGDSNNRAAGHRPGDHTRTKKIEGPRSSSKKNNNAARVVPV
MSTRG.20771.1    ------------------------------------------------------------
                                                                             


                       1210      1220      1230      1240      1250      1260
                 =========+=========+=========+=========+=========+=========+
Ec-28_000090     VSTGSAPKRRMTFGGLRSDKTGEVYKEQRRPSFLEDIGRRATEMAEAAAKATRRISIHGN
MSTRG.20771.1    --------RRMTFGGIRSDKRGEVYKAQRRPSFLDEVGRRAIQAAEATAKLARRISIQGN
                         ####################################################


                       1270      1280      1290      1300      1310      1320
                 =========+=========+=========+=========+=========+=========+
Ec-28_000090     NGQGFELVAIGDRKQSTHETGRRKSAKKGGKD-AGENSSGSDHSENETHFYNRRRHVFEP
MSTRG.20771.1    NGQGFELVAIRDRKESAKNTAARKSAREGGRGGDGENSSDSELCENQTHFYNRTRHVFEP
                 ##############################    ##########################


                       1330      1340      1350      1360      1370      1380
                 =========+=========+=========+=========+=========+=========+
Ec-28_000090     QHTESAPSAWWIRTIGVINLLCMAAYLWWRITRSLKGVDHIIWAFIFLSAECIMAIGMIV
MSTRG.20771.1    QHMEIAPSAWWIRTCGVVNLICMAAYLWWRCMRSLIGVRHIIWAYIFLTAECIMAIGMIV
                 ############################################################


                       1390      1400      1410      1420      1430      1440
                 =========+=========+=========+=========+=========+=========+
Ec-28_000090     GHSSRSFPAHREKVYMDDLTDIDEAIGALKVAVLIPTCGEKTAVMLKALFGNLQLRLWKS
MSTRG.20771.1    GHSSRSFPVHREKVYMDDLTEIDDAIGALKVTVLVPTCGEKTAVMLKALFGNLQLRLWKS
                 ############################################################


                       1450      1460      1470      1480      1490      1500
                 =========+=========+=========+=========+=========+=========+
Ec-28_000090     KNARRDTLRILVLDEKRREEVHKLVSLVYTLSEVVLDKTVREILMREGVAPISAKGFYEH
MSTRG.20771.1    RNARRETLRIVVLDEKRREEVQKLVSLVYTLAEVVLDKIVREILMREGVAPISAKGFYDH
                 ############################################################


                       1510      1520      1530      1540      1550      1560
                 =========+=========+=========+=========+=========+=========+
Ec-28_000090     FANGEHGQRMYDDVNFIRGIEVVSEIDKLIAENDDNISRFSPSVAISRIKERARRASCFD
MSTRG.20771.1    FANGQNGQRMYDDVNFIRGIEIVAEIDKLIAANDDTVSRC--------------------
                 #######################################                     


                       1570      1580      1590      1600      1610      1620
                 =========+=========+=========+=========+=========+=========+
Ec-28_000090     RKEIQPGQKKVWNRNKYIPTIVYYSRIDAGQPRISPKAGNMNRAIFSFNPQEEPLIGEAA
MSTRG.20771.1    ------------------------------------------------------------
                                                                             


                       1630      1640      1650      1660      1670      1680
                 =========+=========+=========+=========+=========+=========+
Ec-28_000090     VIVINDVRHELYPEFLQRTVPYFFTFDKPRRCYKWANIAFIQTPQRFHDRTDWNDPDPLG
MSTRG.20771.1    ------------------------------------------------------------
                                                                             


                       1690      1700      1710      1720      1730      1740
                 =========+=========+=========+=========+=========+=========+
Ec-28_000090     NQAATQFDIVNSGRDGAGGALSCGQGSVWRVQVLRDGIRPDGTKFVKKGMPEDQVGQQGG
MSTRG.20771.1    ------------------------------------------------------------
                                                                             


                       1750      1760      1770      1780      1790      1800
                 =========+=========+=========+=========+=========+=========+
Ec-28_000090     LGFRAEVLIEDTHTSIDLFRQGWKSVYVNFPNERLACCTLPPDTVKWRWKQVLRWHQGAV
MSTRG.20771.1    ------------------------------------------------------------
                                                                             


                       1810      1820      1830      1840      1850      1860
                 =========+=========+=========+=========+=========+=========+
Ec-28_000090     QLAMWKGWGYAVLGENWGTTFQKVFAFDAVSYFLQAFAGEILLIFPIVYGFTNSAPFNTW
MSTRG.20771.1    ------------------------------------------------------------
                                                                             


                       1870      1880      1890      1900
                 =========+=========+=========+=========+=
Ec-28_000090     NIEFALYFFPFIITGVLPTVAALGWQKTPSAKVAFNTPPSK
MSTRG.20771.1    -----------------------------------------
```

```
Parameters used
Minimum Number Of Sequences For A Conserved Position: 2
Minimum Number Of Sequences For A Flanking Position: 2
Maximum Number Of Contiguous Nonconserved Positions: 8
Minimum Length Of A Block: 10
Allowed Gap Positions: None
Use Similarity Matrices: Yes
```

```
Flank positions of the 2 selected block(s)
Flanks: [1209  1290]  [1295  1539]  

New number of positions in Ec-28_000090.aa.fasta.trans.aa_ali.fasta-gb:  327  (17% of the original 1901 positions)
```
